# Functionally distinct T-helper cell phenotypes predict resistance to different types of parasites in a wild mammal

**DOI:** 10.1038/s41598-022-07149-9

**Published:** 2022-02-24

**Authors:** Yolanda Corripio-Miyar, Adam Hayward, Hannah Lemon, Amy R. Sweeny, Xavier Bal, Fiona Kenyon, Jill G. Pilkington, Josephine M. Pemberton, Daniel H. Nussey, Tom N. McNeilly

**Affiliations:** 1 Moredun Research Institute, Pentlands Science Park, Bush Loan, Midlothian, EH26 0PZ UK; 2 Institute of Evolutionary Biology, School of Biological Sciences, University of Edinburgh, Edinburgh, EH9 3FL UK

**Keywords:** ELISA, Immunological techniques, Parasitic infection, Ecology, Immunology, Cytokines

## Abstract

The adaptive immune system is critical to an effective response to infection in vertebrates, with T-helper (Th) cells pivotal in orchestrating these responses. In natural populations where co-infections are the norm, different Th responses are likely to play an important role in maintaining host health and fitness, a relationship which remains poorly understood in wild animals. In this study, we characterised variation in functionally distinct Th responses in a wild population of Soay sheep by enumerating cells expressing Th-subset specific transcription factors and quantifying Th-associated cytokines. We tested the prediction that raised Th1 and Th2 responses should predict reduced apicomplexan and helminth parasite burdens, respectively. All measures of Th-associated cytokine production increased with age, while Th17- and regulatory Th-associated cytokine production increased more rapidly with age in males than females. Independent of age, sex, and each other, IL-4 and Gata3 negatively predicted gastro-intestinal nematode faecal egg count, while IFN-γ negatively predicted coccidian faecal oocyst count. Our results provide important support from outside the laboratory that Th1 and Th2 responses predict resistance to different kinds of parasites, and illustrate how harnessing specific reagents and tools from laboratory immunology will illuminate our understanding of host-parasite interactions in the wild.

## Introduction

The vertebrate immune system is highly complex and composed of many different cell types with different functional roles in responses to infection^1,2^. Variation in the relative abundance of different immune cell types and their responsiveness to stimulation has major implications for infection risk and disease outcomes^3^. The immune system is thought to play an important role in the evolutionary and ecological dynamics of wild populations, protecting individuals from a diverse array of pathogens. Despite this, a shortcoming of many field studies of immunity to date has been the inability to quantify variation in these functionally different cell types, often relying instead on generic measures of immunity, such as skin swelling or natural antibody responses to novel antigen challenge^4,5^. This has largely been due to the lack of suitable immunological tools in non-model systems which would enable the abundance and functionality of immune cells to be quantified. Recent studies applying tools developed for laboratory rodents to their wild counterparts illustrate striking differences between the immune phenotypes of wild and captive animals^1^, as well as variation in diverse aspects of immunity among and within natural populations^6^. At the same time, studies in wild mammals highlight the potential for functional constraints on immune-mediated resistance to different types of parasites to influence patterns of infection and disease dynamics^7^. In order to determine how natural selection has shaped immune responses in wild populations, we first need to characterise variation in functionally-relevant aspects of the immune response. Here, we use expertise and reagents from veterinary immunology to examine variation in functionally distinct arms of the adaptive immune response and test how this variation predicts abundance of gastrointestinal parasites in wild Soay sheep (*Ovis aries*)^8,9^.

The immune system in vertebrates broadly consists of the innate and adaptive arms^10^. Innate responses are generally non-specific and are rapidly activated immediately after parasite antigens are encountered, while adaptive responses are slower to develop but are highly antigen-specific due to the recognition of specific antigens by receptors on B and T lymphocytes. Following antigen-specific activation, B and T cells undergo clonal expansion to generate effector cells that control the immediate infection, and memory cells that provide long-lasting immune protection^10,11^. The adaptive immune system is further divided based on effector functions into humoral immunity, mediated by antibodies produced by B cells, and cellular immunity such as that mediated by cytotoxic T cells and phagocytes^10,11^. Functional diversity of the adaptive immune system is coordinated by CD4^+^ T helper (Th) cells, which can elicit functionally-distinct types of response which support protection of the host against different kinds of challenges^12,13^.

**Figure 1:**
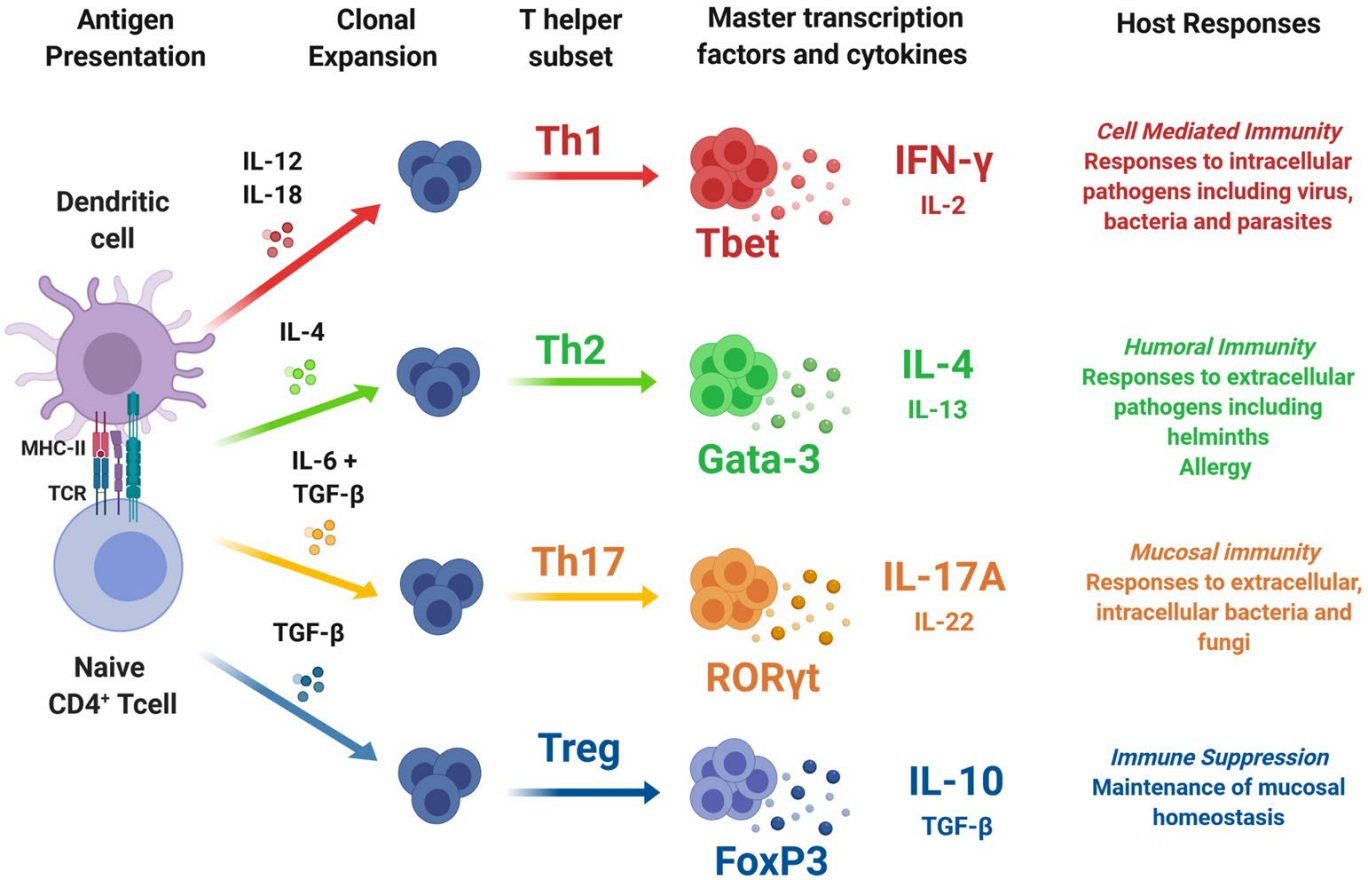
Overview of T helper subsets. Upon encountering foreign antigens, dendritic cells (highly specialised antigen presenting cells) process and present fragments of antigen to naïve CD4^+^ T cells via major histocompatibility complex (MHC) class II molecules. During this process, specific cytokines drive differentiation and clonal expansion of CD4^+^ T cells into functionally distinct T helper (Th) subsets. Each Th subset is associated with a master transcription factor, and secretes specific cytokines involved in coordinating different types of host immune response^12,19–24^.

The main types and functions of Th-mediated immune responses—Th types 1, 2, 17 (Th1, Th2, Th17) and regulatory T cell (Treg) responses—are illustrated in Fig. 1. Th polarisation is largely decided during the early stages of activation of naïve CD4^+^ T cells, when dendritic cells and other innate cells ‘sense’ specific pathogen molecules and, through cytokine and other signalling events, trigger unique ‘master’ transcription factors that become lineage-specific markers for the different Th subsets (Fig. 1; Refs.^14–16^). Each Th subset then secretes a specific group of cytokines that promotes different types of adaptive immune responses, whilst also inhibiting the development of other Th subsets^17,18^. Thus, a broad understanding of adaptive immune function can obtained by quantifying the master transcription factors expressed by Th cells and the cytokines released upon activation (Fig. 1).

In the wild, vertebrates must deal with challenges from a wide range of infectious agents, and co-infection is the norm^25^. As such, there is good reason to expect that variation in Th responses plays a vital role in maintaining health and fitness under natural conditions. In particular, co-infection with intracellular microparasites (e.g. viruses, bacteria, apicomplexans) and macroparasites (e.g. helminths) are expected to elicit trade-offs between host commitment to Th1 vs. Th2 immune responses^2^. For example, in laboratory studies, the greater the suppressive effect of worm infection on Th1 cytokines, the greater the associated increase in microparasite density^26^. Furthermore, recent studies of wild buffalo show that experimental removal of helminths promotes a Th1 response, with downstream consequences for host resistance to intracellular parasites^27,28^. However, measurement of functional T cell responses in wild vertebrates is challenging and has rarely been undertaken. As Fig. 1 illustrates, measuring Th phenotype requires quantification of the number of T cells of different functional types and their functional cytokine response to stimulation. This demands both the immunological tools for T cell phenotyping, which are lacking for most non-model systems, and protocols for either immediate deployment of assays on live cells in the field or preservation of cells for later analysis^4^. Despite these difficulties, recent studies have begun to explore variation in T cell immunity in wild mammals^28–32^. However, to date, with the exception of some studies in humans^33^, no study in wild mammalian populations has quantified patterns of variation in the full range of Th phenotypes or tested their associations with parasite infection.

Here, we characterise Th responses in wild Soay sheep (*Ovis aries*) on St Kilda, using immunological reagents developed for domestic sheep to quantify Th1, Th2, Th17 and Treg responses through activation-specific cytokine release and expression of Th-associated transcription factors. The Soay sheep on St Kilda are principally infected with two groups of gastrointestinal parasites: nematodes and coccidian apicomplexans^8,34^. It is generally accepted that resistance to these parasites groups is broadly associated with different Th responses: Th2 for worms, and Th1 for coccidia^35,36^, and that, in laboratory settings at least, Th1 and Th2 responses are generally antagonistic^17,18^. We examine the associations among different measures of the four main Th response types and their relationship with age and sex, before testing the predictions that: (1) worm burdens should be reduced in animals with stronger Th2 responses, (2) coccidia burdens should be reduced in animals with stronger Th1 responses, and (3) Th1 and Th2 responses should be negatively correlated.

## Results

**Figure 2:**
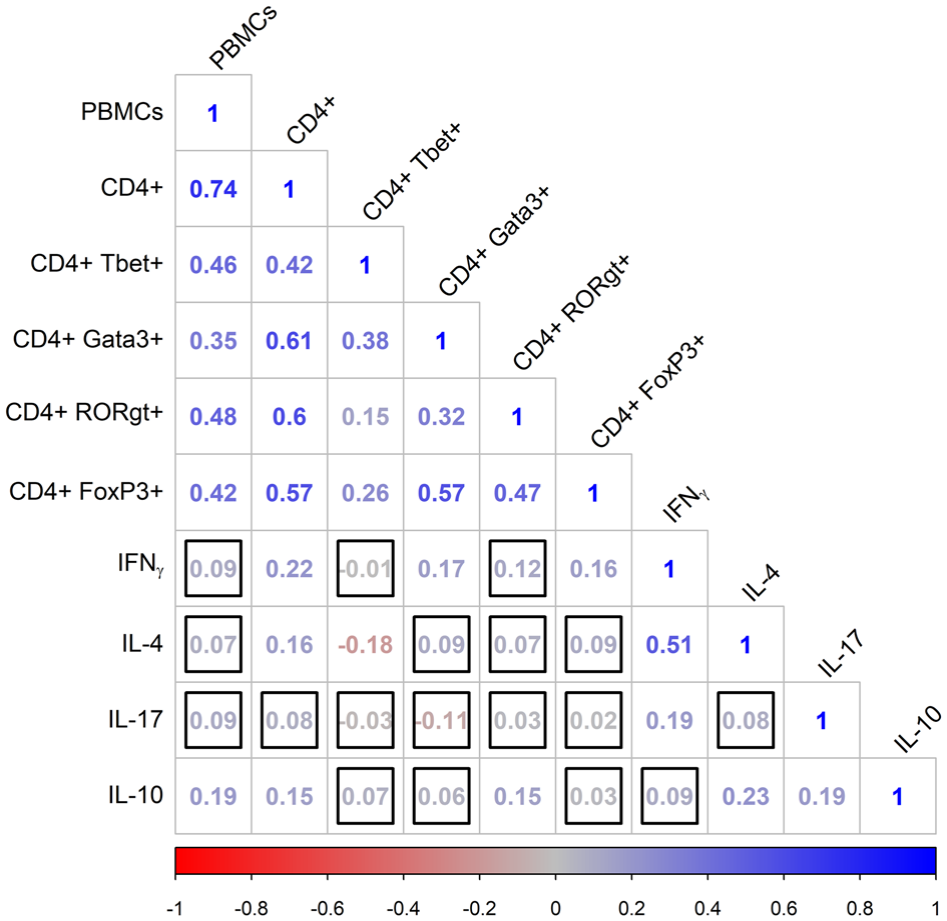
Correlation matrix showing Spearman’s rank correlations between pairs of immunological variables corrected for age and sex, where redder values indicate increasingly negative associations and bluer values indicate increasingly positive associations. Cell phenotypes represent cell counts per ml blood. Correlations outlined with a black square were not statistically significant at α = 0.05.

The correlations between the ten immune variables, after accounting for age and sex differences, are summarised in Fig. 2. The correlations were mostly positive, with the strongest associations observed between the different cell counts and between IFN-γ and IL-4. In general, cytokines were only weakly associated with cell counts. To explore the possibility that the strong positive associations between cell phenotypes was driven by variation in total PBMC numbers, we re-ran the correlation analysis with the proportion of cells of each type within the total leukocyte population rather than the absolute numbers of cells. Since some cells do not express any of the measured markers, these proportions would not necessarily be negatively associated. We found that, in general, associations between cell phenotypes expressed as proportions were relatively weak, suggesting that indeed the strong positive associations were driven by variation in cell numbers (Fig. 2 and Supplementary Fig. S2).

The first principal component of our immune variables explained 32% of the variation and the second explained 25%; the subsequent axes explained 11% or less (Supplementary Table S2). The first axis had strong negative loadings with all of the cell count variables, while the second axis had strong negative loadings with all four cytokines (Supplementary Fig. S3). Plotting our data on the first two principal components (PCs) revealed little evidence of differentiation between the sexes based on the first two PCs, but small differences with age were apparent (Supplementary Fig. S3). Specifically, older animals had lower values of PC2, suggesting stronger cytokine responses with age. This multivariate variation with age but not sex was supported by PERMANOVA analysis: while there was no evidence for an interaction between age category and sex (*F*_*DF*=*3*_ = 0.34, P = 0.901) or the main effect of sex (*F*_*DF*=*1*_ = 0.47, P = 0.592), there was some evidence for variation with age category (*F*_*DF*=*3*_ = 3.54, P = 0.006). Age category, however, only explained a small proportion of the overall variation (R^2^ = 0.06).

**Figure 3:**
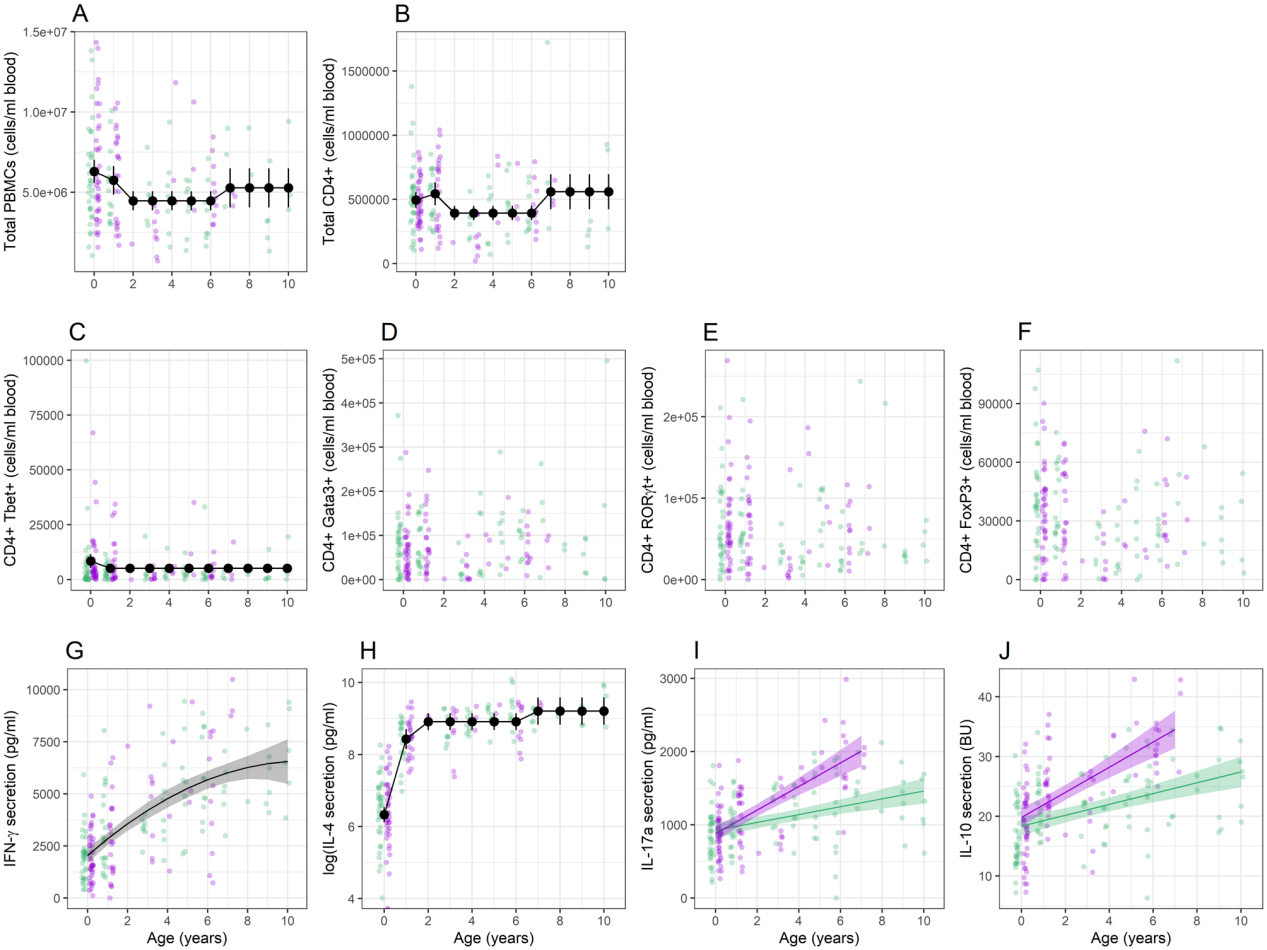
Age- and/or sex-specific variation in (**A**) PMBC; (**B**) CD4^+^ cells; (**C**) CD4^+^Tbet^+^ cells; (**D**) CD4^+^Gata3^+^ cells; (**E**) CD4^+^ RORγt^+^ cells; (**F**) CD4^+^FoxP3^+^ cells; (**G**) IFN-γ; (**H**) IL-4; (**I**) IL-17A; and (**J**) IL-10. Points show raw data, with green representing females and purple showing males. Large points connected by lines show estimates ± 95% CI from a model where age was fitted as a factor (with 2 or 4 levels) and lines with shaded areas show estimates ± 95% CI from models where age was fitted as a continuous variable; green lines represent females and purple lines males. For model details, see Supplementary Tables S4 and S5.

This pattern of sex- and age-specific variation was also apparent in our individual analyses of each immunological variable (Supplementary Tables S3 and S4). Variation in the number of PBMC and CD4^+^ cells was best explained by age as a four-level factor and both appeared to be at their lowest levels in adults compared to the other age groups (Fig. 3A,B). The best-supported model for CD4^+^Tbet^+^ cells suggested higher cell numbers in lambs than in other age classes (Fig. 3C), but there was no evidence for variation with either age or sex in CD4^+^ cells expressing the other three transcription factors (Fig. 3D–F). Finally, all of the cytokines varied with age and/or sex, with a broad trend for increases with age and, where there were sex differences, greater increases in males than females. IFN-γ followed a quadratic trajectory with age, with a steep increase from younger ages to around age four, followed by a shallower increase thereafter (Fig. 3G). IL-4 followed a similar pattern, although the best-supported model had age as a factor with four levels: while IL-4 was low in lambs, it increased dramatically in yearlings, with subsequently smaller increases in adults and then geriatrics (Fig. 3H). The best-supported models for both IL-17A and IL-10 suggested an interaction between age and sex, with linear increases with age in both sexes, but a particularly pronounced increase in males compared to females (Fig. 3I,J).

**Figure 4:**
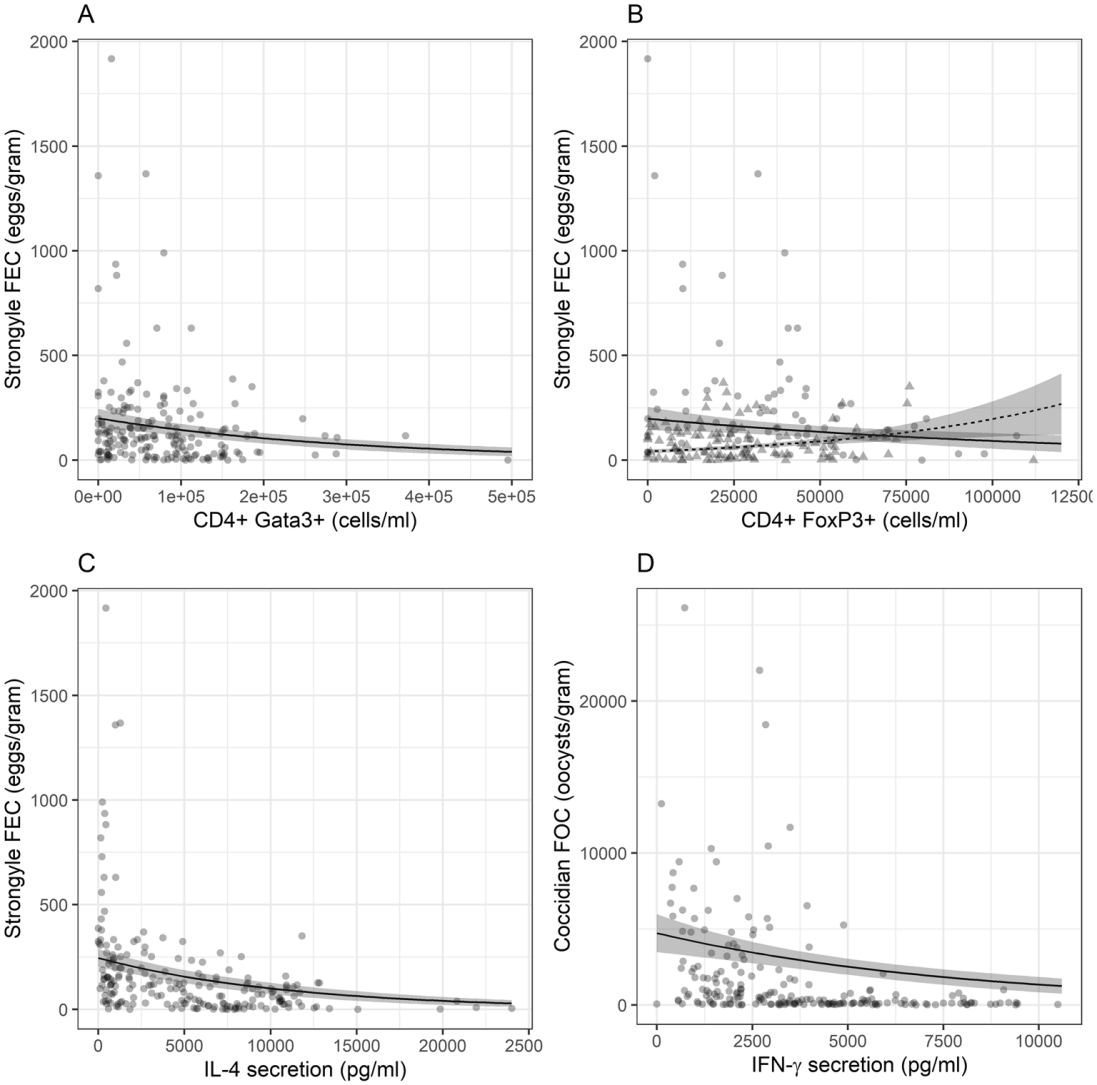
Associations between strongyle faecal egg count (FEC) and (**A**) total CD4^+^Gata3^+^ cells; (**B**) total CD4^+^Foxp3^+^ cells; (**C**) IL-4 secretion; and between coccidian faecal oocyst count (FOC) and (**D**) IFN-γ secretion. Points show raw data and lines show predictions from models in Table 1. In (**B**), lambs are represented by circles and broken lines, adults by triangles and unbroken lines.

As predicted, models of strongyle FEC identified negative associations with Th2-related immune measures while models of coccidian FOC identified negative associations with Th1-related measures (Table 1). In separate models testing the association between FEC and each immune measure, we found that the number of CD4^+^Gata3^+^ cells (estimate = − 2.69E−06 ± 1.07E−06, χ^2^_1_ = 5.42, P = 0.020) and the expression of IL-4 (estimate = − 9.84E−05 ± 2.26E−05, χ^2^_1_ = 16.97, P < 0.001; Table 1) each negatively predicted FEC. We also found significant interactions between age (lamb versus older) and total PBMC, CD4^+^RORγt^+^ and CD4^+^FoxP3^+^ cell counts (Table 1). In each case, there was a stronger negative association with FEC in lambs, and little evidence of an association in adults. We then fitted all of these terms into a single GLM of FEC to determine which were independently significant. Only the main effects of CD4^+^Gata3^+^ (estimate = − 3.24E−06 ± 1.21E−06, χ^2^_1_ = 4.77, P = 0.029; Supplementary Table S6) and IL-4 (estimate = − 8.97E−05 ± 2.27E−05, χ^2^_1_ = 13.81, P < 0.001; Supplementary Table S6), and the interaction between age and CD4^+^FoxP3^+^ were still supported in this model (Supplementary Table S6). Inclusion of coccidian FOC in this final model did not meaningfully alter these results, and FOC did not significantly predict FEC independent of age, sex and the including immune parameters (Supplementary Table S6). Both the number of CD4^+^Gata3^+^ cells and levels of IL-4 were independently negatively associated with FEC, while the interaction between age class and CD4^+^FoxP3^+^ suggested a negative association in lambs but not adults (Fig. 4A–C). In separate models testing the association between FOC and each immune measure, both IFN-γ and IL-4 were negatively associated with FEC (Table 1). However, when we fitted both of these into the same model, IFN-γ was still supported (estimate = − 1.26E−04 ± 4.94E−05, χ^2^ = 6.03, P = 0.014, Fig. 4D) but IL-4 was not (estimate = 1.03E−05 ± 3.16E−05, χ^2^ = 0.12, P = 0.727). Inclusion of strongyle FEC in this model did not meaningfully impact the results (IFN-γ: estimate = − 1.22E−04 ± 4.92E−05, χ^2^ = 5.67, P = 0.018; IL-4: estimate = − 1.28E−05 ± 3.16E−05, χ^2^ = 0.19, P = 0.66) and FEC was not significant (estimate = 5.60E−04 ± 3.51E−04, χ^2^ = 1.40, P = 0.23).

**Table 1:** Results of generalised linear model analysis of associations between either strongyle faecal egg count (FEC) or coccidian faecal oocyst count (FOC) and each of our immunological parameters. Estimates and test statistics are shown for models where only the variable indicted with included in the model; interaction estimates show difference in slope between lambs and adults. Associations significant at α = 0.05 are highlighted in bold.

Trait	N	Strongyle FEC				Coccidian FOC			
		Estimate	SE	χ^2^_1_	P	Estimate	SE	χ^2^_1_	P
**Main effects**									
PBMC	183	− 3.30E−08	2.78E−08	1.17	0.280	3.74E−08	2.92E−08	1.52	0.217
CD4^+^	183	− 5.17E−07	3.06E−07	2.03	0.154	5.10E−07	3.21E−07	2.05	0.152
CD4^+^ Tbet^+^	183	− 1.09E−05	6.86E−06	2.16	0.142	− 1.15E−05	7.19E−06	1.86	0.173
CD4^+^ Gata3^+^	183	**− 2.69E−06**	**1.07E−06**	**5.42**	**0.020**	− 5.48E−07	1.13E−06	0.15	0.701
CD4^+^ RORγt^+^	183	− 5.00E−07	1.53E−06	0.09	0.768	8.28E−07	1.61E−06	0.21	0.648
CD4^+^ Foxp3^+^	183	− 3.26E−06	3.40E−06	0.82	0.364	3.99E−06	3.57E−06	0.87	0.350
IFN-γ	203	− 6.49E−05	3.58E−05	3.63	0.057	**− 0.0001**	**3.63E−05**	**10.35**	**0.001**
IL-4	203	**− 9.84E−05**	**2.26E−05**	**16.97**	**0.000**	**− 4.28E−05**	**2.35E−05**	**4.51**	**0.034**
IL-17	203	3.68E−05	0.0002	0.05	0.818	− 0.0001	0.0002	0.39	0.532
IL-10	203	− 0.003	0.0121	0.06	0.807	− 0.0171	0.0123	1.61	0.204
**Interactions with age**									
PBMC	183	**1.21E−07**	**5.51E−08**	**4.19**	**0.041**	9.03E−08	5.81E−08	2.22	0.137
CD4^+^	183	7.94E−07	6.50E−07	1.08	0.300	7.26E−07	6.82E−07	0.89	0.345
CD4^+^ Tbet^+^	183	2.91E−05	1.54E−05	3.81	0.051	− 2.29E−07	1.64E−05	0.00	0.989
CD4^+^ Gata3^+^	183	3.39E−06	2.22E−06	2.29	0.130	− 1.50E−06	2.37E−06	0.26	0.611
CD4^+^ RORγt^+^	183	**9.54E−06**	**3.07E−06**	**5.85**	**0.016**	5.42E−06	3.25E−06	2.25	0.133
CD4^+^ Foxp3^+^	183	**2.23E−05**	**6.70E−06**	**9.70**	**0.002**	9.27E−07	7.18E−06	0.01	0.915
IFN-γ	203	5.91E−05	1.13E−04	0.30	0.585	− 1.52E−06	0.0001	0.00	0.989
IL-4	203	7.22E−02	5.19E−02	1.70	0.192	− 8.74E−05	0.0002	0.44	0.505
IL-17	203	− 4.52E−04	0.0004	1.38	0.240	7.97E−06	0.0004	0.00	0.983
IL-10	203	− 0.0209	0.0258	0.73	0.394	0.0233	0.0264	0.71	0.399

Statistical significant values are in bold.

## Discussion

We characterised variation in Th responses in a wild population of Soay sheep by enumerating cells expressing Th-specific transcription factors and measuring levels of canonical Th cytokines following ex vivo T cell stimulation. Th cell counts and ex vivo cytokine production were weakly correlated, with only the latter showing variation with age and independently predicting both strongyle and coccidian parasite burdens. We found support for the predictions that increased Th1 responsiveness (IFN-γ) would negatively associate with coccidian FOC, while increased Th2 responsiveness (IL-4) would negatively associate with strongyle FEC. However, contrary to our predictions, we found positive rather than negative associations between Th1- and Th2-associated measures. This provides rare support from outside the laboratory for the importance of different Th subsets for resistance to different kinds of parasite, but adds to mounting evidence from natural systems that Th1/Th2 trade-offs observed in laboratory experiments may not readily translate to natural systems^29,32^.

Age-specific variation in immunity is widely observed and expected: the immaturity of the immune system in juveniles is often associated with higher parasite burden and less effective responses^37^ and this is certainly true of domestic sheep infected with strongyle nematodes^38,39^. In later adulthood, immunosenescence is often detected in wild vertebrates, particularly in adaptive responses^40^. Increases in circulating strongyle-specific antibody levels between lambs and adults in the Soay sheep have previously been observed^41^, as have senescent declines in later life that are associated with increased risk of mortality^42^. Another study in Soay sheep reported pronounced declines with age in the proportions of T cell sub-types in separate cross-sectional studies, most notably naïve (CD45RA^+^) helper (CD4^+^) T cells^43,44^. However, these expected changes were based on fundamental processes in immune development, such as thymic involution, and did not tease apart different functional Th subsets^43,44^. Here, we observed pronounced increases from lambs to adults in ex vivo T cell cytokine responses associated with all Th subsets but little evidence of age-dependence in the number of T cells expressing Th-specific transcription factors. As animals age and are exposed to parasite antigens, the expansion of Th memory pools induces a faster and more rapid cytokine response following activation^45^, potentially explaining why Th cell counts are static but cytokine responses increase with age.

Increases in cytokine responses with age have been previously described in healthy human populations^46^, where a Th1 polarisation can be observed with age^47^. We observed no signs of such a polarisation: production of cytokines associated with Th1, Th2, Th17 and Treg responses all increased with age. However, most human studies focus on cytokine production by CD4^+^ and CD8^+^ cells, whereas our assays potentially include cytokine production by all circulating leukocytes. It is known that activated B cells and natural killer cells can produce IL-10 and IFN-γ, respectively, and may be contributing to the cytokine responses we measured^48,49^. Although a more focused study of the cytokines produced by CD4^+^ T cells might have shown different results, this lack of a shift towards Th1 responses with age could also reflect the consistent life-long exposure to gastrointestinal parasites experienced by wild Soay sheep, which strongly polarise towards Th2 and regulatory responses mediated by IL-10^50^.

We found little evidence for sex differences in Th phenotypes, although ex vivo production of Th17 and Treg cytokines did increase more rapidly with age in males than females. Sex-specific variation in defence against infection is predicted and has been observed in wild populations^51^, with males often exhibiting increased parasite burden and less effective immune responses than females. Previous work on the Soay sheep has shown sex differences in anti-strongyle antibody levels, with males showing weaker responses^52,53^ and higher burdens of nematode parasites across age groups^34,54^. In other wild mammals, male voles exhibited lower levels of expression of the Th2-associated transcription factor Gata3 in peripheral blood and wild male badgers showed lower IFN-γ responses than females following stimulation with PWM, although no sex differences were found in wild buffalo^27,29,31^. Generally, our data provide limited evidence for an important role of Th cell responsiveness underpinning sex differences in parasitism and immune responsiveness.

We found a strong positive association between the Th1 and Th2 cytokines, IFN-γ and IL-4. Antagonism between Th1 and Th2 responses are well-established in laboratory immunology^18,55^, and experimental studies of wild buffalo in which helminth parasites were removed with anthelmintic drugs demonstrated an increase in Th1-associated IFN-γ production^28^. However, data from unmanipulated wild rodent populations provide evidence for synergistic, rather than antagonistic, associations between Th1 and Th2 phenotypes^29,32^, and work in domestic sheep indicates a complex interplay between Th1 and Th2 immune responses is associated with gastro-intestinal parasite resistance^56^. Variation in resource acquisition, common in natural systems and resulting in individual differences in the resources available to invest in immunity, could drive positive associations between different arms of the immune response even if functional trade-offs are present^29,57^. Additionally, challenge from a variety of parasites and the need for considerable plasticity in immune responses, could also lead to selection for the ability to mount effective immune responses to different parasites. As such, trade-offs between different arms of the Th response could be masked by variation in resource acquisition, coinfection, and the need to adjust responses in responses to ecological and epidemiological conditions.

In line with our predictions, levels of the Th2-associated cytokine IL-4 were negatively associated with strongyle FEC, while the Th1-associated cytokine IFN-γ was negatively associated with coccidian FOC. This represents important support for the paradigm that Th1 and Th2 responses have distinct roles in tackling microparasite and macroparasite infections, respectively, from a wild population. In addition, numbers of CD4^+^ T cells expressing Gata3 or Foxp3 (i.e. Th2 and Treg polarised cells) were negatively associated with strongyle FEC. While the association with Gata3 provides further evidence that Th2 immunity is important for control of strongyles, the association between Treg numbers and strongyle FEC, only evident in lambs, is more counterintuitive, given that Treg induction has been suggested as an immune evasion strategy for these parasites^58^. However, the relationship between Treg and helminth immunity is complex—in mice it has been shown that while too many Treg cells impair Th2 immunity and lead to chronic infections, too few lead to dysregulated immune responses and increased worm burdens^59^. Similar findings have also been reported in sheep, with resistance to strongyle parasites being associated with an early Treg-Th2 immune response during primary infection^56^, suggesting that an optimal balance between Th2 and Treg responses is critical to induce effective anti-strongyle immunity, at least during initial exposure to the parasite.

Previous studies of wild mammals have provided evidence in support of associations between Th phenotypes and measures of parasite burden. In a wild badger population, “excretors” of bovine tuberculosis (bTB; *Mycobacterium bovis*) had lower mean IFN-γ responses than bTB-negative individuals^60^. In wild buffalo, anthelminthic-treated individuals had stronger IFN-γ responses and while treatment was not associated with bTB status, treated animals were less likely to die from bTB, suggesting a protective role for Th1 immunity in bTB infection^28^. Finally, expression of Gata3 was positively associated with an index of macroparasite burden in wild adult voles, but negatively correlated in juvenile voles, suggesting a Th2-mediated switch from resistance to tolerance with age^30^. Similarly, domestic sheep lines artificially selected to be more resistant to helminth infection showed enhanced IL-4 (Th2) responses^61–63^. Relatively little is known about coccidian immunity in domestic sheep, although there is convincing evidence that natural infection drives expression of IFN-γ and other Th-1 associated cytokines such as IL-2 and TNF-α^36^. Previous studies in wild Soay sheep found that strongyle FEC was positively associated with coccidian FOC^8^, although we found no evidence for significant associations in our smaller data set. Furthermore, significant associations between strongyle FEC and Th2-associated immune measures were independent of coccidian FOC, just as the associations between coccidian FOC and Th1-associated immune measures were independent of strongyle FEC. Thus the picture in our system appears to be one of variation in the ability to mount responses to diverse parasite taxa, with individuals able to respond effectively to strongyles also being able to respond effectively to coccidia. Our previous work on Soay sheep clearly shows that functionally distinct immune phenotypes (antibodies of different isotypes) can be positively correlated but show different patterns of association with fitness^41^. A crucial next step for our understanding of natural selection on Th phenotype is to relate the variation in different functional measures to fitness in wild populations, and determine whether associations are mediated by effects on parasite burden or individual condition.

## Conclusions

Overall, our results provide important evidence that measuring immune phenotypes associated with different functional arms of the Th response can help us to predict the parasite burden of wild mammals. Whilst ours is not the first study to provide such, we have assessed Th phenotype in an unusually comprehensive fashion, using reagents developed for veterinary immunology. Our results provide evidence simultaneously linking raised Th1-associated responses to reduced microparasite burdens and raised Th2-associated responses to reduced macroparasite burdens in the wild. Whilst this supports the paradigm that different Th arms have effector functions that protect from particular parasite groups, our data challenge the idea that investment in one Th arm constrains investment in another in the wild. One limitation of our study is its cross-sectional nature: longitudinal data is required to determine whether age-related variation is driven by within-individual change or selective effects and how such variation impacts on demography^64^. The small number of longitudinal studies measuring Th-related phenotypes in the wild suggest they are weakly to moderately repeatable within individuals^29,31,65^. Indeed, higher repeatability of IFN-γ in wild buffalo has been shown to predict higher risk of bTB infection, suggesting that the variation on adaptive immune responses could benefit the host by protecting it from infection^65^. Further studies examining longitudinal changes across functional Th subsets and relating these to parasite pressure and demographic rates are likely to illuminate the causes and consequences of variation in Th function for natural populations.

## Materials and methods

### Field and laboratory data collection

Soay sheep have lived unmanaged for several thousand years in the St Kilda archipelago, 65 km west of the Outer Hebrides, Scotland (57° 49′ N, 08° 34′ W). In the 1930s, following the evacuation of the human population from Hirta, the largest island in the archipelago, 107 sheep were moved from the smaller island of Soay onto Hirta^66^. This population has grown to cover the whole island, with numbers only limited by food availability. Since 1985, sheep in the Village Bay area of Hirta (around one third of the total population) have been the subject of an individual-based study. Every year, > 95% of lambs born in the study area are captured within a few weeks of birth (March–May), given an individual identifying ear tag, weighed and blood‐ and tissue‐sampled for genetic analysis. Each August, ~ 50% of sheep are captured in order to collect data on variables including weight and morphometrics alongside faecal and blood samples. Samples used in this study were collected from 238 animals captured in August 2019.

### Ethics

All samples were collected in accordance with relevant guidelines and regulations from the Home Office under project license number PP4825594 and were approved by the University of Edinburgh School of Biological Sciences Ethics committee. This study was carried out in compliance with the ARRIVE guidelines (https://arriveguidelines.org).

### Whole blood stimulation assays

Blood was collected from sheep by jugular venepuncture into lithium heparin vacutainers (Greiner Bio-One International GmbH) and stored at 4 °C prior to being processed, 24 h post capture.

Whole blood stimulations from 208 animals were carried out by mixing 1 ml of whole blood with 1 ml of tissue culture media [RPMI-1640 supplemented with 10% Foetal Bovine Serum (FBS) and 50 μM 2-mercaptoethanol, 2 mM l-glutamine, 100 U/ml Penicillin and 100 μg/ml Streptomycin, 5 μg/ml Gentamicin (all from Sigma-Aldrich, UK)] containing 10 μg/ml of poke weed mitogen (PWM, Sigma-Aldrich, UK) or the same volume of PBS into 15 ml sterile tissue culture tubes (Fisher). Following incubation at 37 °C for 48 h, samples were centrifuged at 300×*g* for 5 min. Supernatants were collected and stored at – 20 °C until assayed for cytokine production.

Capture ELISAs were performed to quantify the secretion of selected cytokines representing different Th subsets: interferon (IFN)-γ (Th1), interleukin (IL)-4 (Th2), IL-17A (Th17) and IL-10 (Treg), following stimulation with PWM. All incubations were carried out at room temperature unless stated otherwise. IL-4 and IFN-γ were quantified using commercial ELISA kits according to the manufacturer’s instructions (MABTECH AB, Augustendalsvägen, SE, Sweden). For the quantification of IL-17A, polyclonal rabbit anti-bovine IL-17A antibodies were used alongside bovine recombinant protein (Kingfisher Biotech, Inc., St. Paul, MN). Mouse monoclonal anti-bovine IL-10 capture and detection antibodies (clones CC318 and CC320b respectively, BioRad) and standard curves produced using supernatants from COS-7 cells transfected with bovine IL-10^67,68^ were used to quantify IL-10 secretion. Washing steps for all ELISAs were performed 6 times with 350 μl washing buffer (Phosphate Buffered Saline (PBS) + 0.05% Tween 20) using a Thermo Scientific Wellwash™ Versa (ThermoFisher). High-binding capacity ELISA plates (Immunlon™ 2HB 96-well microtiter plates, ThermoFisher) were incubated with coating antibodies overnight at 4 °C. Plates were then washed and blocked for 1 h with PBS containing 0.05% Tween 20 (Sigma, UK) and 0.1% Bovine Serum Albumin (BSA, Sigma, UK) for IL-4, IFN-γ and IL-17A or PBS containing 3% of BSA for IL-10. Following a further washing step, 50 μl of supernatants or standards were added in duplicate for 1 h. Subsequently, plates were washed and detection antibodies added for 1 h. This was followed by washing and addition of Streptavidin-HRP (Dako, Agilent, Santa Clara, US) for 45 min. After the final washing step, SureBlue TMB substrate (Insight Biotechnology, London, UK) was added and the reaction was stopped by the addition of TMB stop solution (Insight Biotechnology, London, UK). Absorbance values were read at O.D. 450 nm. In order to quantify the cytokines of interest, samples were analysed 1:20, 1:4, neat or 1:4 for IFN-γ, IL-4, IL-17A and IL-10 respectively. Standard curves were included in all plates and were constructed using 7 serial dilutions of recombinant cytokines ranging from 6.25 to 400 pg/ml for IFN-γ (MABTECH AB); 31.25 to 2,000 pg/ml for IL-4 (MABTECH AB); 23.43 to 1,500 pg/ml for IL-17A (Kingfisher) and 0.206 to 13.2 BU/ml for IL-10^68^. Finally, and in order to account for the non-specific, natural cytokine release in all samples, we subtracted the concentration obtained from the control sample (stimulated with PBS rather than PWM) from the concentration from the PWM stimulated samples, and multiplied the result by the cytokine’s corresponding dilution factor.

### Leukocyte quantification and flow cytometry analysis

To quantify the leukocytes present in each blood sample, following gentle mixing of heparinised blood, leukocytes were enumerated using a Nucleocounter NC-200 (ChemoMetec, Denmark). Briefly, 20 μl of heparinised blood were mixed with 180 μl of Solution 17 (ChemoMetec) and heated at 37 °C for 10 min. The sample was then mixed to obtain a homogenous suspension and loaded into a Via1-Cassette™ (ChemoMetec) prior to counting on a Nucleocounter NC-200 cell counter. Total leukocyte counts per ml blood were recorded for each blood sample and used for the calculation of total cell counts expressing each of the transcription factors investigated.

Multiple colour flow cytometric analysis was carried out as follows. An aliquot of 2 ml of blood was incubated with 10 ml of warm red blood cell (RBC) lysis buffer (1.5 M NH_4_Cl, 100 mM NaHCO_3_, 10 mM N_2_ EDTA in ddH_2_O) for 2 min or until lysis was complete. Following two washes with PBS, Zombie Violet™ Fixable dead cell stain (Biolegend, US) was added to all samples and incubated for 15 min at RT in the dark. Cells were then washed with PBS at 300×*g* for 5 min and stained with the cell surface antibody CD4 labelled to Alexa Fluor^®^ 647 at pre-optimised concentrations for 20 min at RT in the dark (see Supplementary Table S1 for antibody details). Cells were then washed twice with FACS buffer (PBS + 5%FBS + 0.05%NaN_3_) and fixed with Fix/Perm buffer for 30 min at 4 °C (FoxP3 Staining Buffer Set buffer, Miltenyi Biotec, Bergisch Gladbach, Germany) according to manufacturer’s protocol. Following two washes with FACS buffer, cells were re-suspended in 1 ml of PBS and stored at 4 °C. Samples were then transferred to Moredun Research Institute (MRI) and analysed for the expression of Th-specific transcription factors within a month of sample collection. Following permeabilisation, monoclonal antibodies specific for the following Th-associated transcription factors Tbet (Th1), Gata3 (Th2), RORγt (Th17), and FoxP3 (Treg) alongside Isotype control antibody, all conjugated to phycoerythrin (PE), were added to samples and incubated for 30 min in the dark at 4 °C (see Supplementary Table S1 for antibody details). Following staining, cells were washed twice with Permeabilization buffer and analysed immediately. A minimum of 100,000 events were acquired using a Sony SA3800 Spectral Analyzer (Sony Biotechnology, Ltd) and analysed using FlowJo vX for Windows 7. The transcription factor antibodies used for tbet, Gata-3 and RORγt were previously validated using transfected CHO cell lines expressing rbo-tbet, rbo-GATA-3 and rov-RORγt, as well as bovine and ovine PBMC (unpublished data) following the methodology described by Wattegedera et al.^69^.

In order to calculate the percentage of CD4^+^ and T cells expressing each of the hallmark Th-associated transcription factors (Fig. 1), the following gating strategy was carried out. Following dead cell discrimination based on the staining of the viability dye (Supplementary Fig. S1A), and doublet discrimination performed by plotting the height against the area for forward scatter (FSC-H vs FSC-A; Supplementary Fig. S1B), a leukocyte gate was created which included all white blood cells present (peripheral blood mononuclear cells (PBMC), granulocytes and neutrophils; Supplementary Fig. S1C). PBMC were then gated based on the forward scatter (FSC-A) and side scatter (SSC-A) (Supplementary Fig. S1D) and a CD4 gate created based on the fluorescence minus one (FMO) controls (Supplementary Fig. S1E). Threshold levels which determined positivity of each of the transcription factors in CD4^+^ T cells were also set using FMO controls. Consequently, the data obtained represented the percentage of CD4^+^ T cells in PBMC (Supplementary Fig. S1F) and the percentage of CD4^+^ T cells expressing Tbet, Gata3, RORγt or FoxP3 (Supplementary Fig. S1G–J). These were then expressed as a percentage of the total leukocyte population, and total blood leukocyte count data collected in the field was used to calculate the total numbers of CD4^+^ cells expressing each transcription factor per ml of blood.

### Faecal egg/oocyst counts

Faecal samples were collected rectally and faecal egg counts (FEC) for strongyle nematodes and faecal oocyst counts (FOC) for coccidia were conducted on 1 g samples using a modified salt-flotation technique^70^. These counts represent the most common parasites found in the Soay sheep in St Kilda, with strongyles comprising 6 gastrointestinal nematode species and coccidia comprising 11 *Eimeria* species^34^. Samples were stored anaerobically at 4 °C until processed in our laboratory at MRI, around 2 weeks post collection. Briefly, 2 g of faecal material was homogenised with 20 ml of tap water. A subsample of 10 ml was filtered through a sieve into a beaker and washed with 5 ml of fresh water. Following transferring of the filtrate to a 15 ml tube, samples were centrifuged at 200×*g* for 2 min. Supernatant was discarded, pellets resuspended into 10 ml of saturated NaCl solution and centrifuged at 200×*g* for 2 min. Subsequently, tubes were clamped below the meniscus using forceps and parasite eggs/oocysts present in the surface of the saturated NaCl solution were transferred into a cuvette, filled with NaCl solution and parasite eggs/oocysts counted under a microscope.

### Statistical analysis

Out of 238 samples collected, 211 cytokine assays (IFN-γ, IL-4, IL-17A and IL-10) and 188 flow cytometry assays (total PBMCs, CD4^+^, CD4^+^Tbet^+^, CD4^+^Gata3^+^, CD4^+^ RORγt^+^ and CD4^+^FoxP3^+^ cells) were performed. Of the 188 flow cytometry-prepared samples, 2 were not prepared for cytokine assays, leaving 186 samples with all immune measures available. Faecal samples for parasite counts (FEC and FOC) were obtained for 229 sheep and in total 181 sheep had full immunological and parasitological data available (Supplementary Table S2). All analyses were conducted in R ver 3.6.3^71^. First, we assessed the correlations among each of the 10 immune parameters. To ensure that any observed correlations were not due to common age- and sex-related variation among variables, we corrected for age and sex by fitting (Generalised) Linear Models (GLMs) for each variable with sex and age as an 11-level factor (ages 0–10) and their interaction. We assessed the best error distribution for each of the 10 parameters: we fitted linear models to IFN-γ, IL-10 and IL-17A; we fitted linear models to log-transformed IL-4; and fitted negative binomial generalised linear models (GLM) using the “MASS” package^72^ for the other variables (Supplementary Table S4). We used the residuals from these models in our correlation analysis to present age- and sex-corrected results. We estimated Spearman’s rank correlations among all immune measures, assessing statistical significance using the “cor.mtest” function in the R package “corrplot”^73^.

We also explored the dimensionality of the data using principal components analysis (PCA) with the function “prcomp” and used the “adonis” function in the package “vegan”^74^ to run a PERMANOVA analysis in order to test for variation between sexes and age categories across the immune variables. Age was fitted as a four-level categorical variable, with lambs (aged ~ 4 months), yearlings (aged ~ 16 months), adults (aged 2–6 years) and geriatrics (aged 7 +).

We next explored variation in each immunological variables in relation to sex and age (Supplementary Table S2). For cell phenotype traits (PBMC, CD4^+^, Tbet^+^, Gata3^+^, RORγt^+^ and FoxP3^+^ cells) we used a data set of 188 animals where information on all these traits, plus age and sex where available (Supplementary Table S2); for the cytokines, we used a data set of 208 animals (of the 211 animals with cytokine data, 3 were of unknown age; Supplementary Table S2). For each trait, we ran 14 different models with age categorised in different ways in order to best capture age- and sex-specific variation. Error structures were as given above, and as shown in Supplementary Table S4. As well as a null model (0) and a model with sex only (1), we ran models (2–5) with linear age, quadratic age, age as a two-level factor (lambs versus others) and age as a four level factor [lamb, yearling, adult (2–6 years), geriatric (> 6 years)], respectively. We also ran models 2–5 with the added effect of sex (models 6–9) and models 6–9 with the interaction between age and sex (models 10–13). Models for each trait were compared with Akaike Information Criterion (AIC) values, where the lowest value was considered to best fit the data, unless a simpler model had ΔAIC ≤ 2, in which case we selected the simpler model.

Finally, we tested for associations of each of the 10 immune parameters with FEC and FOC using negative binomial GLMs. We used the same data sets as for the analysis with regard to age and sex, minus five animals with missing parasitology data (Supplementary Table S2). We included sex, age as a two-level factor (lambs versus others), and their interaction in all models. We first fitted each of the 10 immune parameters in turn to models of FEC and FOC, and assessed their statistical significance using likelihood ratio tests (LRTs). Next, we fitted interactions between the immune parameters and our two-level age category to test for differences between lambs and adults in associations between immune responses and FEC/FOC. We next fitted all supported immune variables and their interactions into the same GLM in order to determine their independent associations with FEC/FOC. Finally, to determine whether correlations between FEC and FOC could be responsible for associations with immune measures, we tested whether including the other parasite burden measure affected the relationships with immune measures in our final models.

## Supplementary Information

Supplementary Information.

## Data Availability

All data used in this manuscript will be made available online upon final acceptance of the manuscript.
