# Supplementary material for: Functionally distinct T-helper cell phenotypes predict resistance to different types of parasites in a wild mammal
Source: Sci Rep. 2022 Feb 24;12:3197. doi: 10.1038/s41598-022-07149-9 (PMC8873199; doi:10.1038/s41598-022-07149-9)
Supplement: Supplementary file 1 — Supplementary Information. [file 41598_2022_7149_MOESM1_ESM.pdf]

Table S1 *Monoclonal antibodies used for flow cytometric analysis.*

Antigen	***Antibody clone***	***Fluorochrome***	***Isotype***	***Source***
CD4	44.38	Alexa Fluor® 647	IgG2a	BioRad
Isotype control	REA293	PE	Recombinant human IgG1	Miltenyi Biotec
Tbet	REA102/4B10	PE	Recombinant human IgG1	Miltenyi Biotec
Gata3	REA174/TWAJ	PE	Recombinant human IgG1	Miltenyi Biotec
RORγt	REA278/AFKJS-9	PE	Recombinant Human IgG1	Miltenyi Biotec
FoxP3	FJK-16s	PE	Rat IgG2a	eBioscience

Table S2. Sample sizes used in analyses, broken down by *age and sex. "Number sampled" gives the total number of animals that we had any data on; "FEC" gives the number of individuals with known parasite egg/oocyst counts; "Cells" gives the number of individuals with cell phenotype data (PBMC, $CD4^+$, $CD4^+Tbet^+$, $CD4^+Gata3^+$, $CD4^+ROR\gamma t^+$ and $CD4^+FoxP3^+$ cells); "Cytokines" gives the number of individuals with known IFN-γ, IL-4, IL-17A and IL-10 data; "All" gives the number of animals where age, sex, parasite counts, cell phenotype and cytokines were measured.*

**Sex**	**Age**	**Number sampled**	**FEC**	**Cells**	**Cytokines**	**All**
Female	0	39	37	34	37	32
Female	1	23	22	18	21	17
Female	3	10	10	6	8	6
Female	4	10	10	7	8	7
Female	5	9	9	8	9	8
Female	6	13	13	8	11	8
Female	7	9	9	4	6	4
Female	8	2	2	2	2	2
Female	9	7	7	5	6	5
Female	10	8	8	4	6	4
Female	Unknown	4	4	0	3	0
Male	0	49	47	41	43	38
Male	1	24	24	23	23	22
Male	2	1	1	1	1	1
Male	3	11	11	8	8	8
Male	4	3	3	3	3	3
Male	5	3	3	3	3	3
Male	6	10	10	10	10	10
Male	7	3	3	3	3	3
Female	All	134	131	96	117	93
Male	All	104	102	92	94	88
	Known	234	229	188	208	181
	Total	238	233	188	211	181

Table S3. *Results of principal components analysis (PCA) of 10 immunological variables, showing the standard deviation and proportion of total variation explained by each of the principal components.*

	PC1	PC2	PC3	PC4	PC5	PC6	PC7	PC8	PC9	PC10
Standard deviation	1.80	1.57	1.06	0.98	0.78	0.75	0.65	0.53	0.43	0.41
Proportion of variance	0.32	0.25	0.11	0.10	0.06	0.06	0.04	0.03	0.02	0.02
Cumulative proportion	0.32	0.57	0.68	0.78	0.84	0.90	0.94	0.97	0.98	1.00

Table S4. *AIC values from models testing for age- and sex-specific variation in each of 10 immunological variables. Age(2) and Age(4) refer to age modelled as a categorical variable with 2 or 4 levels respectively. For each trait, the model highlighted in **bold** is the model with the lowest AIC; the model highlighted in grey is the chosen model based on AIC. Where a model simpler than the model with lowest AIC had ΔAIC<2 relative to the model with the lowest AIC, the simpler model was chosen to represent the data. Errors structures are NB = negative binomial; LN = log-normal; G = Gaussian.*

**Variables**	**PBMCs**	**CD4+**	**CD4+Tbet+**	**CD4+Gata3+**	**CD4+RORγt+**	**CD4+FoxP3+**	**IFN-γ**	**IL-4**	**IL-17**	**IL-10**
Error structure	*NB*	*NB*	*NB*	*NB*	*NB*	*NB*	*G*	*LN*	*G*	*G*
Null	6091.66	5188.76	3518.67	**4574.31**	**4520.40**	**4262.99**	3827.57	765.25	3163.19	1410.14
Sex	6092.66	5190.29	3518.65	4574.58	4521.53	4264.91	3826.16	761.66	3164.67	1407.61
Age	6087.14	5190.53	3517.70	4574.88	4521.59	4264.27	3713.62	659.73	3115.54	1366.61
Age²	6085.12	5189.49	3518.66	4576.26	4523.46	4265.05	**3710.29**	621.45	3104.14	1360.00
Age(2)	6086.14	5190.60	**3517.16**	4575.94	4521.72	4264.09	3766.43	577.14	3126.99	1357.28
Age(4)	**6083.53**	**5182.91**	3519.90	4578.68	4523.19	4264.29	3715.47	**568.92**	3114.44	1356.44
Sex+Age	6088.91	5191.88	3518.52	4575.80	4523.01	4266.07	3715.08	661.73	3109.63	1349.24
Sex+Age²	6086.62	5191.12	3519.15	4577.31	4524.80	4266.95	3711.98	623.09	3099.34	1343.88
Sex+Age(2)	6087.58	5192.09	3517.28	4576.35	4522.98	4265.96	3767.46	577.38	3126.14	1346.46
Sex+Age(4)	6085.07	5184.41	3520.64	4579.36	4524.37	4266.21	3717.25	570.43	3109.88	1340.56
Sex*Age	6090.71	5193.85	3520.44	4577.46	4525.01	4268.04	3713.39	659.33	3087.56	**1337.68**
Sex*Age²	6088.36	5192.81	3521.08	4579.19	4526.79	4268.48	3712.79	624.81	**3086.60**	1337.88
Sex*Age(2)	6089.57	5193.69	3519.00	4578.34	4524.98	4267.91	3768.65	579.33	3126.40	1347.13
Sex*Age(4)	6090.63	5189.81	3525.27	4584.56	4530.33	4271.87	3713.02	575.07	3107.73	1339.83

Table S5. *Parameter estimates from the best-supported model of age and sex for each of the 10 immunological variables (model highlighted in grey in Table S4). Age(2) and Age(4) refer to age modelled as a categorical variable with 2 or 4 levels respectively. Further details about the selected models can be found in Table S4.*

**Variable**	**PBMCs**	**CD4+**	**CD4+ Tbet+**	**CD4+ Gata3+**	**CD4+ RORγt+**	**CD4+ FoxP3+**
Intercept	15.6487 (0.0575)	13.1089 (0.0607)	8.9944 (0.1858)	11.2429 (0.0932)	11.0259 (0.0652)	10.3556 (0.0845)
Age(2) - lamb	NA	NA	0.0000 (0.0000)	NA	NA	NA
Age(2) - adult	NA	NA	-0.4454 (0.2397)	NA	NA	NA
Age(4) - lamb	0.0000 (0.0000)	0.0000 (0.0000)	NA	NA	NA	NA
Age(4) - yearling	-0.0729 (0.0967)	0.1143 (0.1021)	NA	NA	NA	NA
Age(4) - adult	-0.3366 (0.0888)	-0.2269 (0.0938)	NA	NA	NA	NA
Age(4) - geriatric	-0.1712 (0.1306)	0.1260 (0.1380)	NA	NA	NA	NA

	**IFN-γ**	**IL-4**	**IL-17**	**IL-10**
Intercept	2092.51 (178.83)	6.3955 (0.1047)	934.01 (52.00)	18.3442 (0.7748)
Sex - female	NA	NA	0.00 (0.00)	0.00 (0.00)
Sex - male	NA	NA	-55.35 (73.13)	1.4443 (1.0896)
Age	819.12 (132.85)	NA	52.54 (11.45)	0.9083 (0.1706)
Age^2	-37.10 (16.08)	NA	NA	NA
Age(2) - lamb	NA	NA	NA	NA
Age(2) - adult	NA	NA	NA	NA
Age(4) - lamb	NA	0.0000 (0.0000)	NA	NA
Age(4) - yearling	NA	2.0255 (0.1758)	NA	NA
Age(4) - adult	NA	2.5151 (0.1592)	NA	NA
Age(4) - geriatric	NA	2.8090 (0.2216)	NA	NA
Sex*Age	NA	NA	108.69 (21.73)	11999 (0.3237)

Table S6. *Parameter estimates for a model containing all terms that were statistically supported explanatory variables of FEC when fitted individually (Table 1), both without (left) and with (right) coccidian faecal oocyst count (FOC) included in model. Estimates are from this full model containing all of the listed terms, while test statistics and p-values are from likelihood ratio tests comparing the full model with a model with the term of interest omitted. Terms in bold are statistically supported independently of the other variables in the model.*

	**Without FOC**				**With FOC**			
**Variable**	**Estimate**	**SE**	χ^2_1	**P**	**Estimate**	**SE**	χ^2_1	**P**
Intercept	6.386	0.300			6.333	0.011		
Age (lamb)	0.000	0.000			0.000	0.000		
Age (adult)	-1.886	0.421			-1.833	0.427		
Sex (female)	0.000	0.000			0.000	0.000		
Sex (male)	0.344	0.233			0.313	0.235		
Age (adult): Sex (male)	0.483	0.298	2.57	0.109	0.515	0.300	2.89	0.089
PBMCs	-4.43E-08	4.08E-08			-4.84E-08	4.08E-08		
$CD4^+RORgt^+$	-2.27E-06	2.69E-06			-1.34E-06	2.71E-06		
$CD4^+FoxP3^+$	-7.69E-06	5.54E-06			-9.22E-06	5.56E06		
**$CD4^+Gata3^+$**	**-3.24E-06**	**1.21E-06**	**4.77**	**0.029**	**-3.18E-06**	**1.20E-06**	**4.70**	**0.030**
**IL-4**	**-8.97E-05**	**2.27E-05**	**13.81**	**<0.001**	**-9.03E-05**	**2.27E-05**	**14.01**	**<0.001**
Age (adult): PBMCs	3.59E-08	6.29E-08	0.33	0.566	3.96E-08	6.28E-08		
Age (adult): $CD4^+RORgt^+$	1.99E-06	3.60E-06	0.21	0.647	1.04E-06	3.61E-06		
**Age (adult): $CD4^+FoxP3^+$**	**2.33E-05**	**7.79E-06**	**7.29**	**0.006**	**2.47E-05**	**7.78E-06**	**7.90**	**0.005**

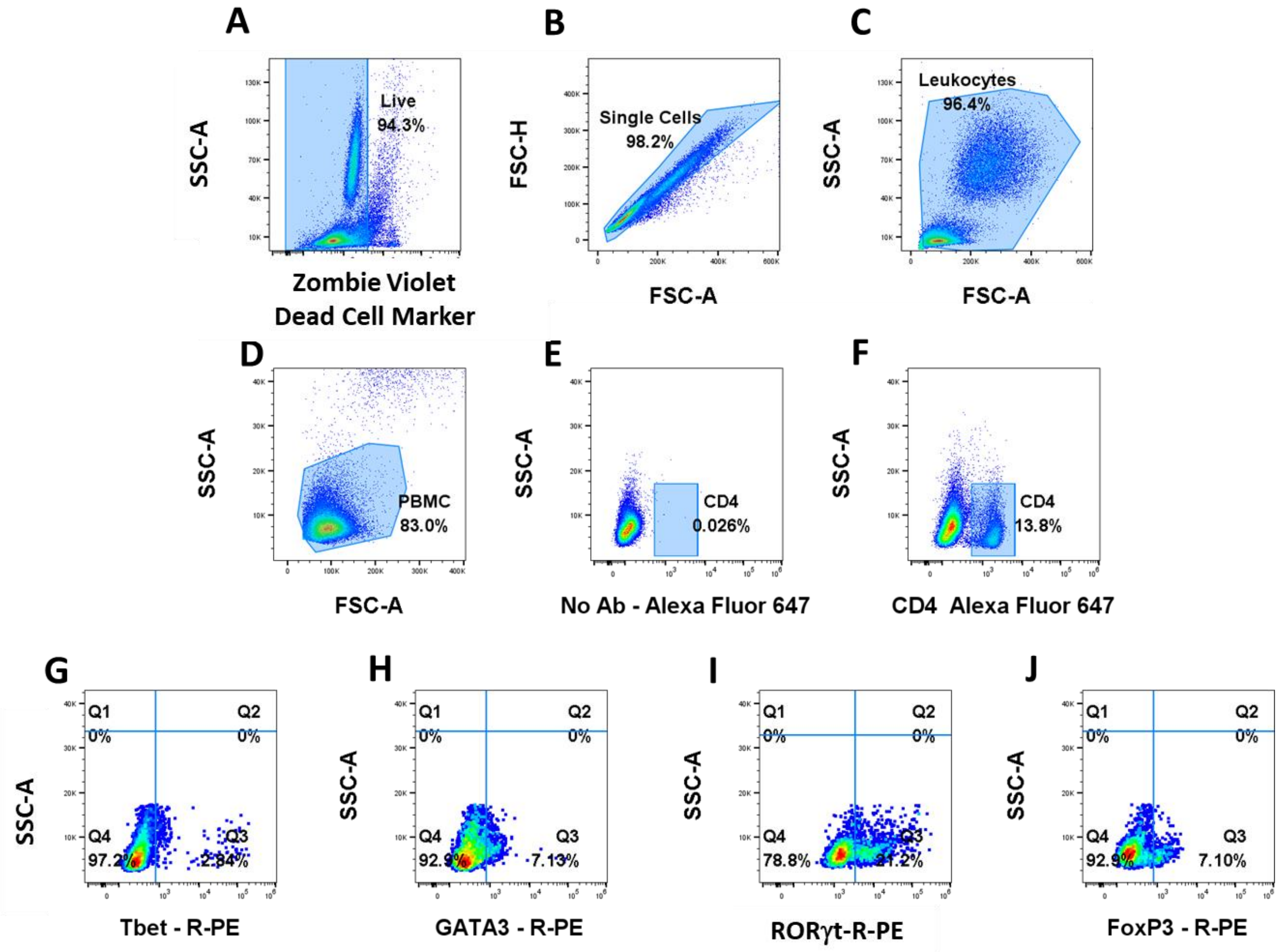

Figure S1. Gating strategy in flow cytometry analysis. *The expression of CD4 and the transcription factors expressed by CD4*$^{+}$ *T cells was studied by multi-colour flow cytometry. Cells were gated to eliminate dead cells (A) and doublets (B). Leukocytes were gated (C) followed by PBMC (D). Expression of CD4 conjugated to Alexa Fluor® 647 (F) in PBMC was determined by the gates set on FMO controls (E). Gates for expression of transcription factors Tbet (G), Gata3 (H), RORγt (I) and FoxP3 (J) all conjugated to R-PE by CD4 were also set using FMO controls. Data shown is from one representative individual with plots G-J corresponding to the expression of transcription factors by CD4*$^{+}$ *T cells. A minimum of 100,000 events were acquired.*

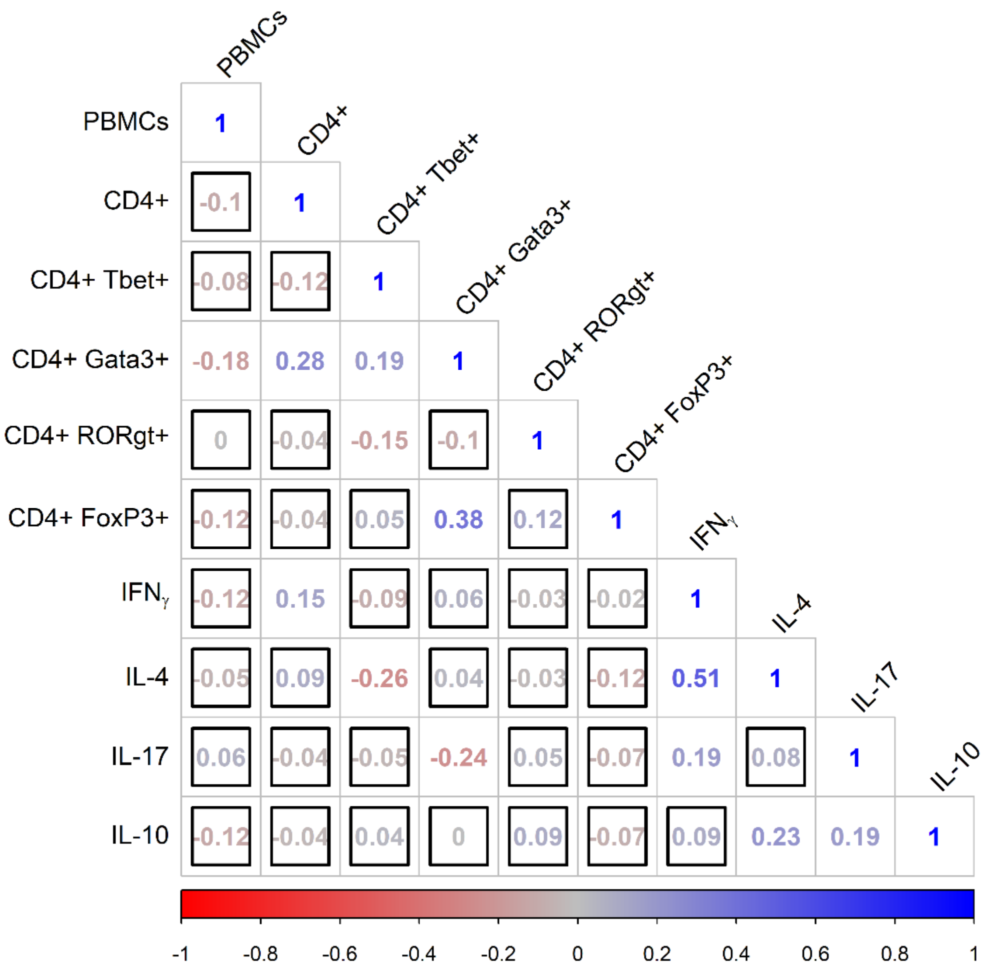

Figure S2. *Correlation matrix showing Spearman's rank correlations between pairs of immunological variables corrected for age and sex, where redder values indicate increasingly negative associations and bluer values indicate increasingly positive associations. Here, cell phenotypes are expressed as percentages of each cell type within the total leukocyte population rather than absolute numbers per ml blood; as such, compare this figure to* ***Figure 2****. Correlations outlined with a black square were not statistically significant at α=0.05.*

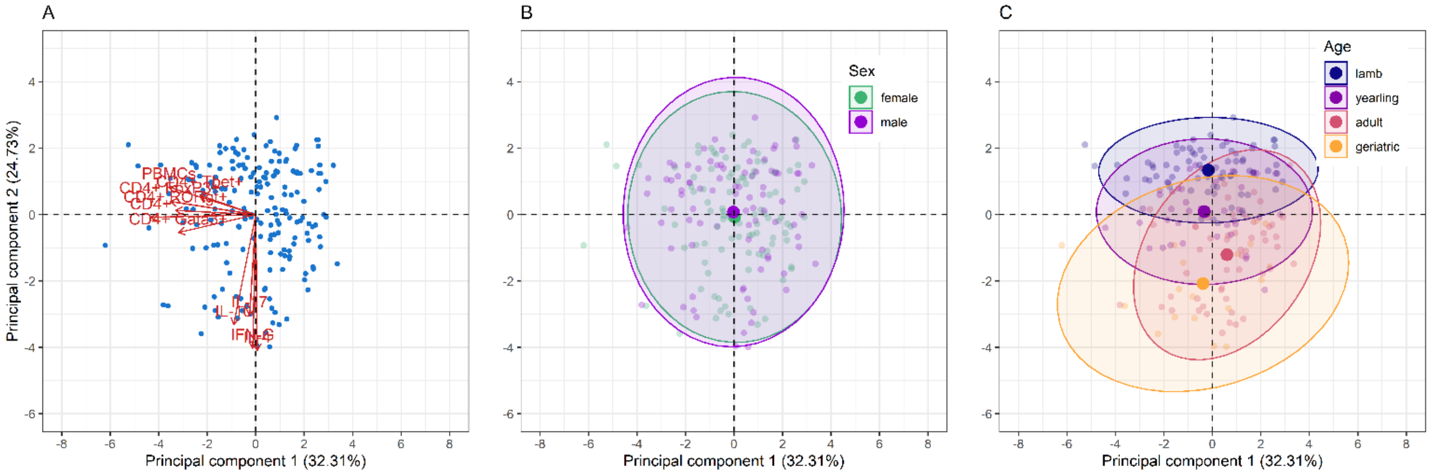

Figure S3. *Biplots showing the results of principal components analysis (PCA) of 10 immunological variables. (A) Arrows show the loading of each variable on the first two principal components, with the length of the vector corresponding to the strength of the influence, while angles between vectors indicate the strength of association between the two variables. (B) and (C) show variation in the placing of data on the first two principal components by sex and age category respectively. Small points show each individual data point; large points show the centre of variation within each sex or age group; ellipses show 95% intervals around this.*
